# Assessing Dietary Diversity in Pregnant Women: Relative Validity of the List-Based and Open Recall Methods

**DOI:** 10.1093/cdn/nzz134

**Published:** 2019-11-18

**Authors:** Phuong Hong Nguyen, Yves Martin-Prevel, Mourad Moursi, Lan Mai Tran, Purnima Menon, Marie T Ruel, Mary Arimond

**Affiliations:** 1 International Food Policy Research Institute, Washington, DC, USA; 2 Nutripass, Université de Montpellier, Research Institute for Development (IRD), SupAgro, Montpellier, France; 3 Alive & Thrive, FHI360, Hanoi, Vietnam; 4 Independent consultant, Washington, DC, USA

**Keywords:** Bangladesh, India, list-based method, open recall method, Minimum Dietary Diversity for Women

## Abstract

**Background:**

The Minimum Dietary Diversity for Women (MMD-W) was validated as a proxy of micronutrient adequacy for nonpregnant women, with proposed data collection being either a list-based or a qualitative open recall method. Few studies have compared the performance of these 2 methods.

**Objectives:**

We compared performance in predicting micronutrient adequacy of food group indicators (FGIs) measured by the list-based and the quantitative open recall methods using varying quantity cut-offs. We also examined the agreement between list-based and open recall FGIs.

**Methods:**

Data were collected in Bangladesh (*n* = 600 pregnant women) and India (*n* = 655). The performance of different indicators to predict micronutrient adequacy was compared using receiver operating characteristic (ROC) analysis. Correlations between list-based and open recall FGIs were calculated using Spearman's rank test; agreement was assessed by the intraclass correlation coefficient (ICC) and kappa statistics. Food groups that were most often misreported by the list-based method were identified.

**Results:**

There were no statistically significant differences in ROC curves between list-based and open recall FGIs in either country. In Bangladesh, correlations between list-based and open recall FGIs varied between 0.6 and 0.8; ICC values were 0.43–0.75; kappa values were 0.51–0.53 when using a cut-off of any quantity or 15 g for open recall, but were lower (k = 0.24) with the cut-off of 1 portion. In India, these values were lower: ∼0.4 for correlation, 0.32–0.37 for ICCs, and 0.17–0.22 for kappas. Food groups most susceptible to misreporting using the list-based method were beans/peas in Bangladesh and other vegetables in India.

**Conclusions:**

Our study provides initial support for the use of list-based questionnaires in assessing food group diversity or prevalence of MDD-W in pregnant women. Additional and context-specific work may be required to understand the potential of simple methodologies to assess consumption of specific food groups. This trial was registered at clinicaltrials.gov as NCT02745249 (Bangladesh) and NCT03378141 (India).

## Introduction

Poor dietary patterns are 1 of the leading risk factors for morbidity and mortality globally, particularly for women and children in low- and middle-income countries (1). However, comprehensive data on diets and diet quality from nationally representative surveys are limited. The lack of indicators to allow for assessment, advocacy, and accountability has been identified as a key constraint to programmatic action to improve diet quality (2, 3). Although several methods for assessing dietary intake are available (4), most of them require highly skilled enumerators and exceptionally resource-intensive data collection, processing, and analysis. Many methods also require the availability of a complete food composition database, the development of which is also resource-intensive. There is a strong and rising demand for simple and feasible, yet accurate, proxy indicators to reflect nutrient adequacy and overall diet quality (5, 6).

In response to this demand, a 10 food-group Minimum Dietary Diversity for Women (MDD-W) indicator was developed and has been validated as a proxy measure for assessing micronutrient adequacy in nonpregnant women at the population level (7). However, questions remain regarding the best and simplest approach for data collection. Both the list-based and open recall methods (8) have certain advantages for measuring dietary diversity. The list-based method demands fewer capacity requirements for enumerators and shorter training time; however, it may be more likely to result in misclassification of foods into food groups or misreporting of some foods, particularly those consumed in small quantities (9). The open recall method is sometimes recommended because it may produce a more accurate and complete recall of all foods and beverages consumed; however, it requires a longer training time and more skillful enumerators who have a reasonable knowledge of local foods (8).

The MDD-W indicator was designed for situations where a quantitative 24-h recall (or other quantitative method) is not feasible, and neither the list-based nor the open recall methods require estimation of portion sizes (the quantity of food consumed). Intuitively, quantity, as well as diversity, matters in achieving micronutrient adequacy, which is reflected, for example, in recommended daily (or weekly) portion sizes in food-based dietary guidelines. However, minimum quantities have rarely been examined in the context of evaluating the performance of simple food group indicators (FGIs). To our knowledge, only 4 studies have examined the effect of different minimum quantity criteria on indicator performance (10–13). Three of these studies demonstrated that a minimum quantity cut-off of 10 g, 15 g, or higher improved indicator performance in predicting micronutrient adequacy in children and women (10, 12, 13), but 1 study of infants and young children aged 6–23 mo showed no difference in indicator performance when using a minimum quantity cut-off of 10 g (11).

To address this gap in the literature, this article uses data from pregnant women in Bangladesh and India to: *1*) compare the performance in assessing micronutrient adequacy of 2 methods of measuring FGIs—the list-based method and the open recall method using varying quantity cut-offs (any quantity, ≥15 g, and ≥1 portion); and *2*) examine the agreement of list-based and open recall FGIs in predicting mean dietary diversity, achievement of MDD-W, and individual food group consumption.

## Methods

### Data source and study population

We used baseline data collected as part of 2 studies that assessed the feasibility of integrating a package of maternal nutrition interventions during pregnancy in existing health systems in Bangladesh (14) and India (15). In Bangladesh, data were collected between June and August 2015 in 20 subdistricts (*upazilas*) from 4 districts (Mymensingh, Rangpur, Kurigram, and Lalmonirhat). In India, data were collected between October and November 2017 in 26 rural blocks from 2 districts (Unnao and Kanpur-Dehat) in the state of Uttar Pradesh. Representative samples were selected from each study site, including 600 pregnant women aged 13–43 y in Bangladesh and 655 pregnant women aged 18–40 y in India.

The studies received ethical approval from the Institutional Review Boards of BRAC University in Bangladesh, the Suraksha Independent Ethics Committee in India, and the International Food Policy Research Institute in the USA. Written informed consent was obtained from all study participants.

### Data collection

We used both qualitative list-based questionnaires (8) and a quantitative open recall method (16) to gather data on intake of different food groups during the day and night before the survey. The 2 methods were administered on the same day, but at different times and by different interviewers.

In the list-based method, the trained enumerator read a list of foods and beverages from each food group to the respondent and asked her if she had consumed any of them during the previous day or night. The qualitative questionnaire was based on a list of 17 food groups in Bangladesh and 19 food groups in India that reflected the distinctive characteristics of food consumption in each country. Several examples of local foods or dishes made from these local foods were provided for each food group. Details of food-group questions from the list-based method are presented in **Supplemental Table 1**.

The open recall method assessed food consumption using a multiple-pass quantitative 24-h recall. In the first 2 passes, the respondent listed and described food items in a manner similar to the qualitative 24-h open recall described in *Minimum Dietary Diversity for Women: A Guide to Measurement* (8). Women were asked to describe all the foods and beverages they consumed during the previous day and night, as well as the time of consumption, cooking method, and portion size. Recipes of composite dishes were recorded by asking the women who had prepared them to show the food ingredients they used. In the third pass of the quantitative recall, the quantities of each food and beverage consumed were estimated, and the amount of each ingredient was measured using an electronic dietary scale with a precision of 2 g; this third pass has no analog in the simple qualitative open recall method described in the guide. Repeated recalls were performed on nonconsecutive days in 10% of the sample; these data were used to adjust for the intraperson variance when estimating usual intakes.

### Calculating FGIs, usual intakes, and probability of adequacy

For the list-based method, the food groups were recategorized into the 10 food groups used in the MDD-W measurement guide (8): *1*) starchy staple foods, *2*) beans and peas, *3*) nuts and seeds, *4*) dairy products (milk, yogurt, and cheese), *5*) flesh foods (meat, fish, poultry, and liver/organ meats), *6*) eggs, *7*) dark green leafy vegetables, *8*) vitamin A-rich fruits and vegetables, *9*) other vegetables, and *10*) other fruits. A list-based FGI was obtained by summing the number of food groups consumed by each woman.

In the open recall method, all food items recorded during the first 2 passes of the quantitative 24-h recall in the survey were grouped into the same 10 food groups (8). These food group variables were used for descriptive analyses of mismatches in reported consumption of the food group. Three open-recall-based FGIs were then derived from the full quantitative recall (including portion size estimation) using 3 criteria for counting a food group: *1*) any quantity consumed, *2*) a minimum quantity consumed of 15 g, or *3*) a minimum of 1 portion, as defined in the respective dietary guidelines of Bangladesh (17) and India (18).

Nutrient intakes were then calculated using food composition tables specific to Bangladesh (19) and India (20). Estimated usual intakes for each woman were used to calculate probabilities of adequacy for individual micronutrients (21, 22). The mean probability of adequacy (MPA) of each woman's intake was computed as the average of the probabilities of adequacy for a set of 11 micronutrients—calcium, iron, zinc, vitamin C, vitamin B1, vitamin B2, niacin, vitamin B6, folate total, vitamin B12, and vitamin A (12).

### Statistical analysis

We used descriptive analyses to report the distribution of FGIs (ordinal scores), based on the qualitative list-based and on the quantitative open recall methods for each country. We performed a receiver operating characteristic (ROC) analysis to compare how well the 2 methods predicted micronutrient adequacy as measured by MPA (using an MPA cut-off of ≥0.60) (7). The area under the ROC curve (AUC) summarizes the predictive power of micronutrient adequacy across all possible cut-off values for the list-based and open recall FGIs. An AUC significantly different from 0.5 and ≥0.70 was considered satisfactory to indicate predictive performance (23, 24). We performed tests of equality of AUCs for different FGIs, adjusting significance levels for multiple tests across classifiers via Sidak's correction. We hypothesized that the open recall FGI using the 1 portion cut-off would have the strongest relation to MPA, so we compared the AUC for the open recall FGI using the 1 portion cut-off against the AUC for other cut-offs (any quantity and 15 g) and for the list-based FGI.

To examine the agreement between list-based FGI and the different open recall FGIs, we first used scatter plots to show the distribution of list-based FGI compared with those of the 3 open recall FGIs (25). We calculated the correlation among FGIs obtained by the 2 methods using Spearman's rank correlation coefficient, which is appropriate because we considered our FGI variables to be ordinal. We then calculated the raw differences in the number of food groups consumed between pairs of measurements collected by the open recall method and list-based method. For agreement among the ordinal list-based and open recall FGIs, we calculated intraclass correlation coefficients (ICCs) and ordinal weighted kappa statistics (26). For agreement among women achieving MDD-W (dichotomous variable), we used simple kappa statistics. The ICC was used to estimate consistency between methods with a higher ICC indicating a higher degree of consistency (27, 28). Kappa scores of 0.21–0.40 indicate fair agreement; 0.41–0.60, moderate agreement; 0.61–0.80, substantial agreement; and 0.81–1.00, almost perfect agreement (29).

Next, we reported the proportion of women who had consumed each of the 10 food groups based on the qualitative list-based or the quantitative open recall methods. We tested the differences between these proportions using an ANOVA test, adjusting significance levels for multiple comparisons across classifiers via Bonferroni's correction. We then calculated the frequency of misreporting for each food group and identified the food groups that were most often misreported by the qualitative list-based method compared with the quantitative open recall method with different cut-offs.

Statistical significance was set at a *P* value of < 0.05 and all tests were 2-sided. Analyses were performed using Stata 15.1 (Statacorp).

## Results

### Comparison of the relative performance of list-based and open recall FGIs to predict micronutrient adequacy of the diet

The performance of FGIs to predict micronutrient adequacy of the diet was similar for the list-based and open recall methods in both countries (AUCs ranged from 0.76 to 0.82 in Bangladesh and from 0.69 to 0.78 in India) (**Table 1**). The small differences among AUCs for the list-based and open recall FGIs were not statistically significant (all *P* > 0.05).

**TABLE 1:** Performance of food group indicators to predict micronutrient adequacy of the diet (mean probability of adequacy >0.60)

	AUC (95% CI)	*P* values for tests of equality of ROC area
Bangladesh		
Open recall FGIs		
≥1 portion	0.82 (0.75, 0.88)	Reference
Any quantity	0.76 (0.70, 0.83)	0.216
≥15 g	0.77 (0.71, 0.83)	0.255
List-based FGI	0.78 (0.71, 0.85)	0.819
India		
Open recall FGIs		
≥1 portion	0.78 (0.72, 0.85)	Reference
Any quantity	0.71 (0.63, 0.79)	0.273
≥15 g	0.74 (0.66, 0.82)	0.693
List-based FGI	0.69 (0.62, 0.76)	0.216

AUC, area under the curve; FGI, food group indicator; ROC, receiver operating characteristic.

### Agreement between FGIs across methods

**FIGURE 1:**
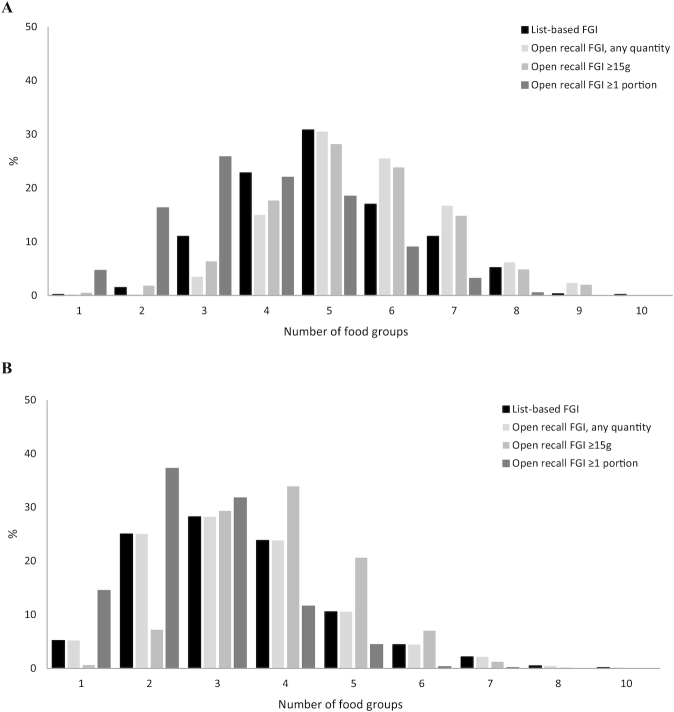
Distribution of food group indicator scores by method and country. Bangladesh (A), India (B). FGI, food group indicator.

The distribution of FGI scores, by method and country, are presented in **Figure 1**. In Bangladesh, the mean list-based FGI was 5.1 ± 1.4, which was lower than the mean open recall FGIs obtained when using any quantity (5.6 ± 1.3) or a 15 g minimum (5.4 ± 1.5), but higher than the mean open recall FGI when using a 1 portion cut-off (3.8 ± 1.5) (**Table 2**). The mean food group consumption in India was ∼1.5 food groups lower than in Bangladesh for both methods (including all 3 open recall cut-offs). The correlations between list-based and open recall FGIs are displayed in **Supplemental Figure 1**. Spearman ρ varied between 0.6 and 0.8 for Bangladesh and was ∼0.4 for India (all *P* < 0.001).

**TABLE 2:** Agreement between food group indicators measured by the list-based method and open recall method with different cut-offs

	Mean ± SD	Median (IQR range)	Mean difference^1^ open recall FGIs − list-based FGI (95% CI)	ICC (95% CI)	% Agreement^2^	Weighted kappa
Bangladesh						
List-based FGI (reference)	5.1 ± 1.4	5.0	—	—	—	—
		(4.0, 6.0)				
Open recall FGIs						
Any quantity	5.6 ± 1.3	6.0	0.57	0.73	91.4	0.51***
		(5.0, 7.0)	(0.50, 0.64)	(0.48, 0.85)		
≥15 g	5.4 ± 1.5	5.0	0.32	0.75	91.7	0.53***
		(4.0, 6.0)	(0.24, 0.39)	(0.68, 0.80)		
≥1 portion	3.8 ± 1.5	4.0	−1.31	0.43	83.8	0.24***
		(3.0, 5.0)	(−1.42, −1.21)	(−0.01, 0.67)		
India						
List-based FGI (reference)	3.3 ± 1.4	3.0	—	—	—	—
		(2.0, 4.0)				
Open recall FGIs						
Any quantity	4.2 ± 1.1	4.0	0.88	0.32	83.7	0.17***
		(3.0, 5.0)	(0.77, 0.98)	(0.11, 0.47)		
≥15 g	3.9 ± 1.1	4.0	0.59	0.37	85.7	0.22***
		(3.0, 5.0)	(0.48, 0.69)	(0.24, 0.48)		
≥1 portion	2.6 ± 1.1	2.0	−0.79	0.33	85.8	0.22***
		(2.0, 3.0)	(−0.89, −0.69)	(0.14, 0.47)		

1 Mean difference compared scores of list-based and open recall methods with different cut-offs.

2 Percent of perfect agreement of food group indicator scores between list-based and open recall methods with different cut-offs.

*** *P* < 0.0001. FGI, food group indicator; ICC, intraclass correlation coefficient.

The mean differences among open recall and list-based FGIs were positive for the open recall FGIs using any quantity and 15 g minimum cut-offs, and negative for the open recall FGI with the 1 portion cut-off (**Table 2**). This indicates underreporting from the list-based method compared with the open recall method with any quantity or 15 g cut-offs, but overreporting compared with the 1 portion cut-off. ICC values ranged from 0.43 to 0.75 for Bangladesh, and from 0.32 to 0.37 for India. The agreement of ordinal FGIs among list-based and open recall methods ranged from 84% to 92%. Weighted kappa statistics indicated moderate agreement (k = 0.51–0.53) in Bangladesh when using any quantity or 15 g cut-offs for open recall FGIs, but only fair agreement with the 1 portion cut-off. In India, the agreement between list-based FGI and open recall FGIs ranged from 84% to 86%, but weighted kappa values were lower (k = 0.17–0.22).

### Agreement of MDD-Ws across methods

The share of women achieving MDD-W (consumed ≥5 food groups) in Bangladesh was 65% according to the list-based FGI; according to the open recall FGIs, it was 81% using the any quantity cut-off, 74% using the 15 g minimum cut-off, and 31% using the 1 portion cut-off. The share was much lower in India (18% for the list-based method). The agreement in classifying women as achieving or not achieving MDD-W was ∼80% in Bangladesh when comparing the open recall FGIs using the any quantity or 15 g cut-offs with the list-based FGI, but the agreement was lower (60%) when comparing the open recall using the 1 portion cut-off with the list-based FGI (**Table 3**). Conversely, in India, agreement was highest between the list-based FGI and the open recall FGI based on 1 portion (83%), with somewhat lower agreement with the 15 g (72%) and any quantity (66%) cut-offs. Simple kappa values for the dichotomous indicators were very similar to weighted kappa values for the ordinal FGIs in the 2 countries.

**TABLE 3:** Agreement among food group indicators measured by list-based and open recall methods for the Minimum Dietary Diversity for Women

	List-based FGI		Agreement statistics	
	<5 food groups *n* (%)	≥5 food groups *n* (%)	% agreement	Simple kappa
Bangladesh				
Open recall FGI—any quantity				
<5 Food groups	103 (17.2)	10 (1.7)	80.0	0.51***
≥5 Food groups	110 (18.3)	377 (62.8)		
Open recall FGI ≥15 g				
<5 Food groups	127 (21.2)	31 (5.2)	80.5	0.55***
≥5 Food groups	86 (14.3)	356 (59.3)		
Open recall FGI ≥1 portion				
<5 Food groups	192 (32.0)	221 (36.8)	59.7	0.27***
≥5 Food groups	21 (3.5)	166 (27.7)		
India				
Open recall FGI—any quantity				
<5 Food groups	360 (55.0)	45 (6.9)	65.8	0.19***
≥5 Food groups	179 (27.3)	71 (10.8)		
Open recall FGI ≥15 g				
<5 Food groups	410 (62.6)	55 (8.4)	71.9	0.23***
≥5 Food groups	129 (19.7)	61 (9.3)		
Open recall FGI ≥1 portion				
<5 Food groups	525 (80.2)	98 (15.0)	82.9	0.18***
≥5 Food groups	14 (2.1)	18 (2.8)		

*** *P* < 0.0001. FGI, food group indicator.

### Misreporting of food groups by the list-based questionnaire relative to the open recall method

Several food groups that made up the FGI scores varied when measured by the list-based and open recall methods, except for starchy staple foods that were consumed by almost all women in the study sample in both countries in the previous 24 h (**Table 4**). In Bangladesh, comparing the list-based approach with the 3 open recall cut-offs, beans and peas were underreported by a large share of respondents (42–58%), whereas flesh foods and vitamin A-rich fruits were overreported by 12–43% and 12–18% of respondents, respectively. The largest proportion of overreporting of some food groups (dairy, flesh foods, eggs, dark green leafy vegetables, and other vegetables) was between the list-based and the open recall methods with a 1 portion cut-off. In India, the picture was different: when comparing the list-based approach to the open recall method with either the any quantity or 15 g cut-off, a large proportion of respondents underreported other vegetables (59–67%), dairy (31%), and other fruits (14–17%), whereas when comparing the list-based and the open recall methods based on 1 portion, the proportion of women who reported consuming these food groups were closer. Overall, the list-based recall for the consumption of nuts, dark green leafy vegetables, and vitamin A-rich fruit and vegetables was higher than the open recall for these groups.

**TABLE 4:** Proportions of pregnant women having consumed food groups in Bangladesh and India, based on list-based and open recall methods

		Open recall FGIs		
	List-based FGI	Any quantity	15 g	1 portion
Bangladesh				
All starchy staple foods	100.0^a^	100.0	100.0^a^	100^a^
Beans and peas	36.2^a^	94.2^b^	83.7^c^	74.7^d^
Nuts and seeds	2.8^a^	6.8^b^	4.8^a^	2.8^a^
Dairy	37.3^a^	34.8^a^	33.5^a^	25.0^b^
Flesh foods	84.2^a^	74.8^b^	73.0^b^	42.0^c^
Egg	25.5^a^	24.2^a^	23.2^a^	7.3^b^
Dark green leafy vegetables	47.2^a^	45.7^a^	45.2^a^	29.3^b^
Vitamin A-rich fruits and vegetables	24.3^a^	20.0^a^	18.8^a^	8.8^b^
Other vegetables	91.5^a^	99.3^b^	94.0^a^	33.0^c^
Other fruits	57.7^a^	63.7^ab^	62.0^ab^	52.5^ac^
India				
All starchy staple foods	98.9^a^	99.9^b^	99.9^b^	99.9^b^
Beans and peas	52.2^a^	56.0^a^	48.7^b^	41.2^c^
Nuts and seeds	23.8^a^	15.7^b^	10.4^c^	6.7^c^
Dairy	54.4^a^	82.6^b^	82.1^b^	51.9^c^
Flesh foods	6.4^a^	4.4^a^	4.4^a^	4.0^a^
Egg	3.2^a^	2.4^a^	2.4^a^	2.3^a^
Dark green leafy vegetables	42.8^a^	37.3^ab^	35.4^b^	16.3^c^
Vitamin A-rich fruits and vegetables	8.6^a^	1.7^b^	1.7^b^	0.2^b^
Other vegetables	24.3^a^	89.8^b^	79.5^c^	19.1^a^
Other fruits	20.2^a^	32.7^b^	28.9^b^	14.2^a^

Serving size for Bangladesh: rice, wheat, lentil: 30 g/serving; dark green leafy vegetables: 125 g; vegetables: 150 g; fruits: 80 g; fish, meat, poultry: 80 g; egg: 60 g; milk: 150 g. Serving size for India: cereal, pulses: 30 g; egg, meat, chicken, fish 50 g; milk, vegetables, fruits: 100 g.

Different letters in superscript in a same row indicate statistically different proportions. FGI, food group indicator.

## Discussion

Our findings showed that, on average, pregnant women in Bangladesh consumed ∼5 food groups and those in India consumed 3.3 food groups, with 65% and 18% achieving MDD-W, respectively (based on the list-based method). Both the list-based FGI and open recall FGIs performed adequately and similarly in predicting micronutrient adequacy of the diet; AUCs ranged from 0.69 to 0.82 and were not statistically significantly different among the various indicators. FGIs and MDD-W indicators measured by the list-based and the quantitative open recall methods had moderate agreement in Bangladesh and fair agreement in India. In both countries, the list-based method tended to misreport the consumption of specific food groups compared with the open recall method, particularly with the 1 portion cut-off.

In Bangladesh, misreporting exceeded 10% for beans and peas, flesh foods, and vitamin A-rich fruits, but was less frequent for other food groups when comparing the list-based FGI and the open recall FGIs using the any quantity or 15 g cut-offs. For other food groups, overreporting was large only when comparing the list-based FGI and the open recall FGI using the 1 portion cut-off. In India, the pattern was less consistent; underreporting was found for dairy products and other vegetables, whereas overreporting was observed for other food groups when comparing the list-based FGI and open recall FGI using the 1 portion cut-off.

We have tried to identify the specific foods that were responsible for the misreporting of food group consumption by looking in more detail at the frequency of specific foods reported with the open recall approach. The underreporting of the beans and peas food group in Bangladesh could be due to foods made of soybeans that were not identified as such (when given as examples to the respondent) on the list-based questionnaire. In India, it could be that milk put in tea was not recorded with the list-based method, and that onions and/or tomatoes were not identified in mixed dishes. This is consistent with findings from Burkina Faso, where misreporting was common for foods used in mixed dishes or in small quantities for sauce ingredients (9). As for overreporting from the list-based method versus open recall with the 1 portion cut-off, note that the MDD-W methodology was not designed to capture minimum quantities of 1 portion but rather only to exclude trivial quantities. This is because there is no simple way to operationalize 1 portion and portion sizes per food group vary substantially across national food-based dietary guidelines; there is no global agreement on portion sizes. The overreporting from the list-based FGI compared with the open recall FGI using the 1 portion minimum was expected, given that 1 portion is likely to be greater than amounts often consumed by respondents, especially of flesh foods, which tend to be expensive and therefore likely consumed in small amounts when available. Overreporting could result if some food groups are reported through the list-based questionnaire because they are easily identifiable (e.g. flesh foods, eggs, some kinds of vegetables) but are consumed in quantities of <1 portion.

The lower correlation and less agreement among list-based and open recall FGIs in India compared with Bangladesh needs further examination. In both countries, we have worked with enumerator teams specially trained in collecting information on dietary intake, with a standardized adaptation process for the list-based questionnaire. The nature of the diet in each country is complex, with several mixed dishes containing multiple ingredients. These were also the focus of special training to ensure that mixed dishes and food items with multiple ingredients were classified in the right food groups. Ingredients with trivial quantities mainly used to add flavor were to be put in the “condiments and seasonings” group.

The MDD-W measurement guide suggests 2 qualitative methods (list-based and open recall) to measure food group diversity (8), but limited evidence existed on the validity of these 2 nonquantitative methods at the time of publication. Our study used an open recall derived from rigorous quantitative 24-h recalls. Although there is currently no true gold standard for dietary assessment, since all recall-based methods entail error, the quantitative 24-h recall is still considered the best and only feasible method for a range of applications, including describing intakes, examining associations, and evaluating the effects of interventions (30). Therefore, our comparison of intakes derived from a list-based questionnaire to those derived from a quantitative 24-h recall could be considered a type of relative validation. Both the list-based and open recall methods in our study covered the same period of recall (i.e. the day and night prior to the survey), thereby preventing differences related to varying time frames.

To our knowledge, this is the first study that uses data from South Asian countries to compare the relative validity of 2 measurement methods for MDD-W in pregnant women. Strengths of the study include our access to 2 data sets with well-documented high-quality training and methods, resulting in high-quality dietary data, and with sufficiently large sample sizes. Ours is also the first study to compare list-based and open recall methods for MDD-W using multiple quantity cut-offs. The need for such studies is urgent, given that there is wide uptake of MDD-W.

Limitations of the study include that our open recall was quantitative, so we are not comparing the 2 methodologies described for MDD-W, where both the open recall and list-based methods are qualitative. However, the first 2 passes of our quantitative recall are similar to a qualitative recall, and our approach enabled us to also look at the issue of varying quantity cut-offs. An additional limitation of our study is that both sites were in South Asia, and ideally studies would incorporate a wider range of geographies and food cultures.

Our AUC results showed similar predictive values among the list-based and the open recall FGIs (even for the different minimum cut-offs), illustrating that the list-based FGI performed as well as the open recall FGIs in predicting micronutrient adequacy of the diet for pregnant women. This has important implications because the list-based method has several advantages, such as lower capacity requirements for enumerators and shorter training time, which in turn reduce the cost of data collection. Our findings confirm the validity of the list-based FGI as a simple tool to measure dietary diversity in pregnant women that may be more feasible than other complex measures in resource-poor settings. Our study thus provides initial support for the use of the list-based FGI in assessing FGIs (ordinal scores) or prevalence of MDD-W. However, for assessing prevalence of intake of specific food groups, estimates from list-based FGI and the open recall FGIs differed quite substantially for select food groups in each country, so additional methodological work from a wider range of contexts is required to understand the potential of using simple methodologies to assess consumption of specific food groups.

## Supplementary Material

nzz134_Supplemental_File

## Notes

Funded by The Bill & Melinda Gates Foundation and the Canadian Department of Foreign Affairs, Trade and Development through Alive & Thrive, managed by FHI360; and the CGIAR Research Program on Agriculture for Nutrition and Health (A4NH), led by the International Food Policy Research Institute.

Author disclosures: PHN, YM-P, MM, LMT, PM, MTR, and MA, no conflicts of interest.

Supplemental Table 1 and Supplemental Figure 1 are available from the “Supplementary data” link in the online posting of the article and from the same link in the online table of contents at https://academic.oup.com/cdn/.

Abbreviations used: FGI, food group indicator; ICC, intraclass correlation coefficient; MDD-W, Minimum Dietary Diversity for Women; MPA, mean probability of adequacy; ROC, receiver operating characteristic.
