# Supplementary material for: Assessing Dietary Diversity in Pregnant Women: Relative Validity of the List-Based and Open Recall Methods
Source: Curr Dev Nutr. 2019 Nov 18;4(1):nzz134. doi: 10.1093/cdn/nzz134 (PMC7101484; doi:10.1093/cdn/nzz134)
Supplement: nzz134_Supplemental_File [file nzz134_supplemental_file.docx]

**Assessing dietary diversity in pregnant women: Relative validity of the list-based and open recall methods**

**Nguyen et al.**

**Online supporting materials**

**Supplemental Table 1: Food list in the List-based methods in Bangladesh and India**

Interviewer: First ask if yesterday was a special day, like a celebration or feast day or a fast day where anyone in the HH ate special foods or where they ate more or less than usual or did not eat because they were fasting?

Yes 1

No 0

Was **yesterday** a special day where special kinds of foods were eaten?

If yesterday was **not** a special day, then ask the respondent about the types of foods that they or anyone else in their household ate yesterday during the day and at night.

If yesterday **was** a special day, then ask the respondent to describe the foods (meals and snacks) consumed the **day before yesterday (or the last normal day)** during the day and night, whether at home or outside the home.

**Now I’d like to ask you to describe everything that you ate or drank yesterday (or the last normal day) during the day or night, whether you ate it at home or anywhere else. Please include all foods and drinks, any snacks or small meals, as well as any main meals. Remember to include all foods you may have eaten while preparing meals or preparing food for others. Please also include food you ate even if it was eaten elsewhere, away from your home. Let’s start with the first food group.**

Did you eat cereals yesterday (examples of cereal include rice, bread made of wheat, puffed rice, pressed rice, noodles, or any other foods rice, wheat, maize/corn)?

|  | **Bangladesh** | **India** |
| --- | --- | --- |
| 1 | CEREALS  Rice, bread made of wheat, puffed rice, pressed rice, noodles, or any other foods rice, wheat, maize/corn, | FOOD MADE FROM GRAINS  (Bread (rotisetc), rice,noodles or other foods made from grains) |
| 2 | VITAMIN A RICH VEGETABLES AND TUBERS  pumpkin, carrots, sweet potatoes that are orange and yellow inside | VITAMIN A RICH VEGETABLES AND TUBERS (Pumpkin, carrots, squash or sweet potatoes that are yellow or orange inside) |
| 3 | WHITE TUBERS AND ROOTS OR OTHER STARCHY FOODS  Potatoes, white yams, white sweet potato (not orange inside), potato crisps or other foods made from roots (not orange or yellow roots) | WHITE ROOTS AND TUBERS AND PLANTAINS  (White potatoes, white yams, raw banana, arbhi, shakarkhandior any other foods made from white-fleshed roots or tubers, or plantains) |
| 4 | DARK GREEN LEAFY VEGETABLES  Dark green leafy vegetables, including spinach, red amaranth leaves, green amaranth, puishak, laushak, kumrashak, kolmishak, mustard leaves, yam leaves, koloishak (pea leaves), methishak (amaranth leaves), dhekishak, demishak etc | DARK GREEN LEAFY VEGETABLES  (List examples of any medium-to-dark green leafy vegetables, including spinach, fenugreek, amaranth, mustard leaves wild/foraged leaves) |
| 5 | OTHER VEGETABLES  Other vegetables (e.g., squash, eggplant, green papaya, cauliflower, cabbage, onion, radish, sheem/borboti (beans) | OTHER VEGETABLES  (Brinjal, cauliflower, onion, radish, beans, gourds etc) |
| 6 | VITAMIN A RICH FRUITS  Ripe mangoes, ripe papaya/pawpaw, jack fruit | VITAMIN A RICH FRUITS **(Ripe mango, ripe papaya)** |
| 7 | OTHER FRUITS (e.g. banana, apples, guava, oranges, other citrus fruits, pine apple, shakalu, watermelon, olives, grapes, jambura (grapefruit) berries, kamranga, tamarind, plum | OTHER FRUITS  Other fruits (e.g. banana, apples, guava, oranges, other citrus fruits etc) |
| 8 | Any beef, goat, lamb, chicken, duck, or other birds, liver, kidney, heart, or other organ meats | MEAt, POULTRY  (chicken, mutton, lamb, etc) |
| 9 | EGGS: Eggs of different birds – chicken, duck, etc.; with yolk, without yolk | EGGS  (Eggs from poultry or any other bird) |
| 10 | FISH: Big/small fresh or dried fish or shellfish (e.g prawn, crab etc.) | FISH |
| 11 | Any foods made from beans, peas, or lentils  beans, peas, lentils, other pulses, soybeans, peas | PULSES (BEANS, PEAS, AND LENTILS)  (Mature beans or peas (fresh or dried seed), lentils or bean/pea products) |
| 12 | NUTS and SEEDS | NUTS AND SEEDS: (Any tree nut, groundnut/peanut or certain seeds, or nut/seed “butters” or pastes) |
| 13 | MILK AND MILK PRODUCTS  Milk, cheese, yogurt or other milk products | MILK AND MILK PRODUCTS: (Milk, cheese, paneer/cottage cheese, yoghurt or other milk products but NOT including butter, ice cream, or sour cream) |
| 14 | OILS AND FATS  Oil, fats or butter added to food or used for cooking including ghee | OILS AND FATS |
| 15 | SWEETS Sugar, molasses, honey, misti, cold drinks, chocolates, candies, biscuits | SWEETS: (Sugary foods, such as Indian sweets, chocolates, candies, cookies/sweet biscuits and cake, sweet pastries or ice cream) |
| 16 | SPICES, CONDIMENTS, BEVERAGES: Spices (cumin, coriander, salt), condiments (pickles, chutney), etc. | CONDIMENTS dried herbs, spices, garlic, onion, chili peppers, ginger root, ect |
| 17 | Tea/Coffee | Sugar-sweetened beverages: (Sweetened fruit juices and “juice drinks”, soft drinks/fizzy drinks, yoghurt drinks or sweet tea or coffee with sugar) |
| 18 |  | Savoury and fried snacks |
| 19 |  | Other vitamin or protein supplements (tablet or powder) |

**Supplemental Figure 1**

**
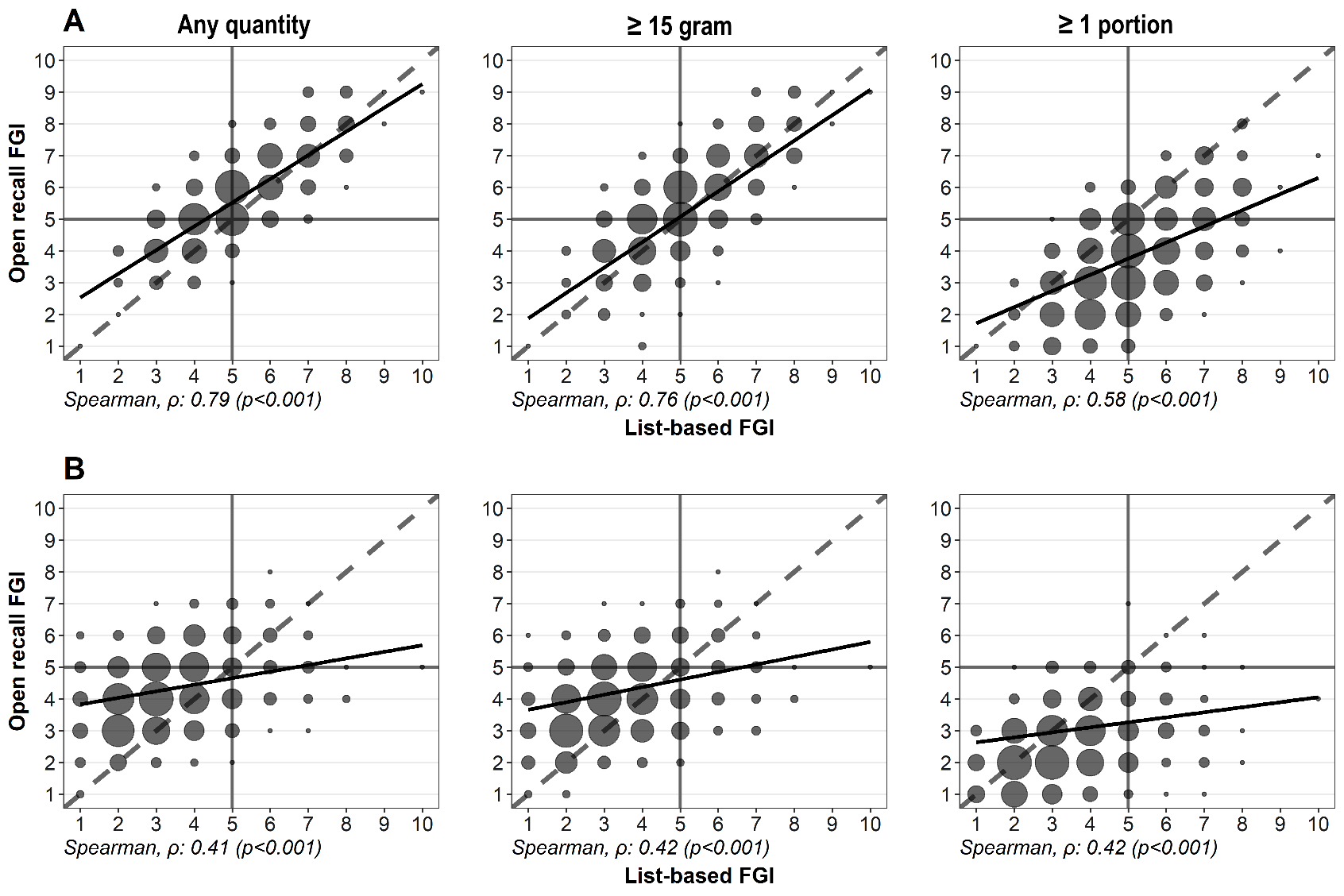
**
